# Raman Spectroscopy Detects Bone Mineral Changes with Aging in Archaeological Human Lumbar Vertebrae from Thornton Abbey

**DOI:** 10.1177/00037028241291601

**Published:** 2024-11-08

**Authors:** Sheona Isobel Shankland, Hugh Willmott, Adam Michael Taylor, Jemma Gillian Kerns

**Affiliations:** 1 Lancaster Medical School, 4396 Lancaster University, Lancaster LA1 4YG, UK; 2 School of History, Philosophy and Digital Humanities, University of Sheffield, Sheffield, UK

**Keywords:** Raman spectroscopy, archaeology, biological, life sciences, biomedical

## Abstract

Archaeological human remains provide key insight into lifestyles, health, and diseases affecting past societies. However, only limited analyses can be conducted without causing damage due to the destructive nature of current technologies. The same problem exists with current clinical analyses of the skeleton, and the preferred advanced imaging techniques only provide macroscopic information. Raman spectroscopy could provide chemical information without detriment to archaeological bone samples and perhaps the need for invasive diagnostic procedures in the future. This study measured archaeological human vertebrae to investigate if chemical differences with aging were detectable with Raman spectroscopy and if differences in mineral chemistry could contribute to information on bone mineral diseases. The three lowest bones of the spine (lumbar vertebrae L3–L5), which are subject to the heaviest loading in life, of nine adults from three age groups (18–25, 25–45, and 45+ years) were provided by the Thornton Abbey Project. Three biomechanically important anatomical locations were selected for analysis; likely sites chosen to measure any chemical changes associated with aging, the vertebral body center and the zygapophyseal joints. Results detected chemical changes associated with aging. These changes relate to the minerals phosphate (∼960 cm^–1^) and carbonate (∼1070 cm^–1^), which are fundamental to bone function. Overall mineralization was found to increase with aging, but while carbonate increased with age, phosphate increased up to ∼45 years and then declined. These fluctuations were found in all three vertebrae, but were more distinct in L5, particularly in the vertebral body, indicating this is an optimal area for detecting bone mineral chemistry changes with aging. This is the first Raman analysis of bone samples from the historically significant site of Thornton Abbey. Results detected age-related changes, illustrating that ancient remains can be used to enhance understanding of modern diseases and provide information on the health and lifestyle of historic individuals.

## Graphical abstract

**Graphical Abstract:**
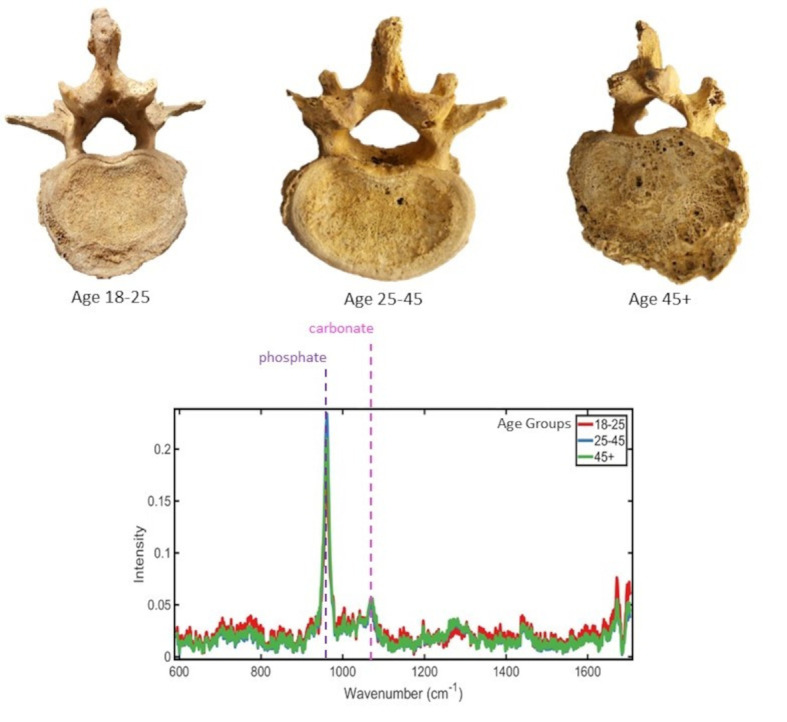
This is a visual representation of the abstract.

## Introduction

**Figure 1.**
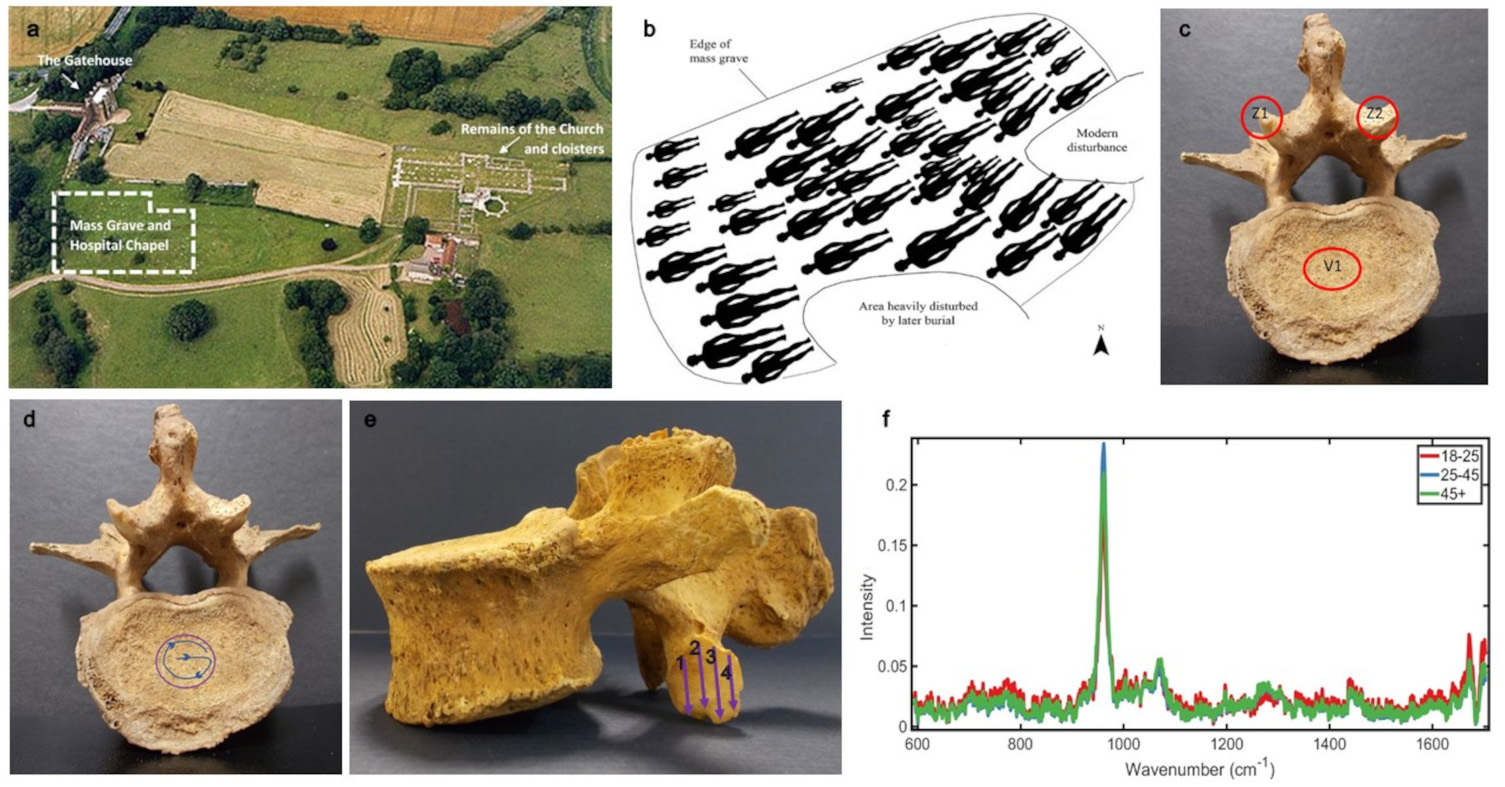
(a) An aerial photograph of Thornton Abbey.^1^ (b) Schematic of the burials interred at the mass grave at Thornton Abbey.^1^ (c) Third lumbar vertebra (L3) showing target areas for measurement using Raman spectroscopy. (d) L3 vertebra showing the V1 target area, and the path data were collected along. (e) Third lumbar vertebra showing Z1/Z2 target area and the path the data were collected along over the joint facet. (f) Example of average spectra. This example shows all processed vertebrae classed by age.

Thornton Abbey was an Augustinian monastery founded in 1139 CE.^1^ The remains of the abbey are located in rural North Lincolnshire, and it is one of the UK's most intact monastic estates (Figure 1a).^2^ The University of Sheffield excavated the site from 2012 to 2016, with a knoll to the south of the gatehouse investigated in 2013. A trench was opened in the northeast corner of the mound revealing articulated human skeletal remains. The grave cut and arrangement of skeletons indicated they were buried in a staggered single event,^3^ a mass grave (Figure 1b).^2^ A confirmed 48 individuals were buried with care during a single interment in eight overlapping rows, with most individuals showing evidence of a being wrapped in a shroud at the time of burial.

The grave is likely a result of the Black Death, known to be present in the area in 1349 CE, supported by molecular evidence of the *Yersinia pestis* bacteria and radiocarbon dating on two sets of remains estimating the grave to 1295–1400 CE (95.4% probability).^2,4–6^ The 48 individuals were placed into seven categories according to estimated age, with 21 categorized as adult (18+). It is from these individuals that this study examined the lower lumbar vertebrae to investigate age-related changes in skeletal chemistry. Archaeological samples enable more comprehensive analyses, permitting selection of areas of biomechanical significance across a whole bone instead of surgically extracted smaller samples from modern donors, which are often elderly or pathological, preventing age-related mineral changes being observed.

The skeleton constantly changes and adapts to the forces acting upon it due to Young's modulus.^7^ Bone has two main components, protein and mineral; both are crucial to function and fluctuate with development, healing, aging, and disease.^8^ Protein gives bones flexibility, and mineral gives bone strength and rigidity. It is a delicate balance keeping the skeleton strong and supportive, but not so rigid that sudden increased force would cause fracture or too soft and cause bending.^8,9^ Alterations to this balance occur naturally as bone remodels;^10^ however, pathologies and changes with aging that, while not necessarily classed as pathological, could be precursory and indicative of future undesirable changes, also occur. The most commonplace is osteoarthritis, which affects approximately half a billion people worldwide,^11^ having huge socioeconomic impact.

The chemistry of these changes at a molecular level is still poorly understood. This investigation uses archaeological material, so chemical analyses of these remains will contribute to a growing body of literature about the individuals interred at this unique medieval site. In addition, they will enhance understanding of changes in chemical minutiae, expanding our knowledge of bone and skeletal development and potentially improving diagnoses and treatment options for skeletal conditions.

Employing Raman spectroscopy, this study aimed to investigate changes in bone mineral chemistry with aging. As these skeletons were underground for over seven centuries, this study did not focus on protein information as it is subject to unpredictable amounts of degradation depending on burial practices and soil environment, affecting preservation.^12^ Raman spectroscopy uses a nonionizing, near-infrared laser to excite the molecules of a sample without causing damage. The vibrations from the energy exchange between sample and laser cause a change in the wavelength of the incident light when it scatters from the sample, a phenomenon called Raman scattering. Different molecules require different amounts of energy to vibrate; therefore, the energy exchange is molecule specific. This allows a biochemical fingerprint to be established as each wavenumber corresponds to identifiable molecules.^13^

This study hopes to improve understanding of healthy bone chemistry and also observe any age-related changes in bone mineral that could be attributed to aging and age-related diseases such as osteoarthritis.^14^

## Experimental

### Materials and Methods

All Thornton Abbey samples were obtained from the University of Sheffield. The lower three lumbar (L) vertebrae (L3–L5) of nine individuals between ∼18 and 45+ years were provided, dating to the middle fourteenth century. These bones were selected as they are the most consistently highly loaded during life, so are often the focus of skeletal clinical assessments, such as a dual-energy X-ray absorptiometry (DEXA) bone density scan,^15^ which only gathers gross skeletal information as opposed to the chemical information gathered in this study. They were categorized into three age groups by osteologists at the Thornton Abbey Project:^2^ 18–25 (youngest) 25–45 (middle), and 45+ (older) (Table S1, Supplemental Material).

Vertebrae were hand cleaned using a dry and then damp brush to remove soil from the surface and ingrained soil, respectively. Samples were air-dried prior to analysis.

### Data Collection

A Renishaw inVia (Renishaw plc, UK) Raman microscope (50× long working distance objective lens, NA 0.5, spectral resolution 0.3 cm^–1^) with a 785 nm laser with a 0.2 mm spot size was used to collect 912 spectra. The instrument was calibrated using silicon and polystyrene. Spectra were acquired using 100% (∼2 mW at sample) laser power for 30 accumulations at 2 s. The spectral range was 1704 to 594 cm^−1^.

### Measurements

Three biomechanically significant areas on each vertebra were selected, which were consistently present on most specimens with intact cortical bone.

The measurement locations (Figure 1c) were the center of the inferior vertebral body (V1), acquired in a “G” pattern to avoid overlap (Figure 1d), and the inferior zygapophyseal joints (Z1 and Z2) acquired across the entire articular surface in columns (Figure 1e).

The V1 measurement marks the center from the widest point mediolaterally and anteroposteriorly, from which a 0.4 cm radius was isolated using a stainless steel ring (stainless steel does not have a Raman signature in the wavelength region of interest).

A minimum of 10 independent spectra were collected from each area, more than 0.2 mm away from any previous measurement to ensure no overlap as the laser spot size is 0.2 mm.

## Data Analysis

Preprocessing and analyses were conducted using Matlab v.2017a with an “irootlab” coding plugin.^16–18^ All spectra were baseline corrected using polynomial order 7 and then vector normalized. The data were analyzed to consider age, vertebral level, and vertebral region, aligning with modern clinical needs (Figure 1c).

Principal component analysis linear discriminant analysis (PCA-LDA) was performed as a combined approach to visualize potential chemical differences among samples by reducing dimensionality while maximizing variance across spectra. This analysis methodology was based on work by Kelly et al.^19^

Performing PCA-LDA in Matlab v.2017a produced scores plots to visualize trends in bone chemistry with 95% confidence intervals applied to two-dimensional (2D) scores plots, and loading plots to show which wavenumbers/chemicals significantly contributed to these trends.^18,19^ One-dimensional (1D) scores plots only contain information along the *x*-axis; separation along the *y*-axis is for interpretive clarity. True peak heights were calculated from the difference between the peak height and the baseline measurements, and the subsequent mineral-to-mineral ratios (carbonate: phosphate) calculated. Mineral crystal maturity was determined by calculating the full width at half-height (FWHH) of the phosphate peak (960 cm^–1^). Raman band intensity changes were also calculated as percentages for additional information on mineral changes. Results allowed the detection of spectral variance which could be linked to pattern changes between and within sample groups.

## Results

All samples are fundamentally still bone and therefore have very similar chemical composition, producing similar spectra. All spectra demonstrated a profile consistent with bone (Figure 1f), with well-defined peaks relating to the inorganic minerals phosphate (∼960 cm^–1^) and carbonate (∼1070 cm^–1^). The loading plots for these analyses detected contributions from multiple peaks within the phosphate band. These are indicative of different phosphate species that contribute to the overall band at ∼960 cm^–1^. These have been previously identified,^20^ but their contributions to overall phosphate, and therefore bone mineral, changes with aging were beyond the scope of this study. Based on these results, phosphate band differences with aging could be explored in future work.

Protein peaks were detected, but were not investigated due to variable protein degradation in archaeological skeletal material. Bone diagenesis in unfossilized remains is a growing area of research, examining the changes in the skeleton after death and the role of endogenous and environmental bacteria.^21^ The inability to assess if changes in protein were a result of aging or burial conditions makes the presence of protein peaks in the analysis unreliable in the assessment of changes in bone with aging in these samples.^22^

Individual N22 from the older group (45+) possessed significant differences in bone mineral chemistry, obscuring other intersample differences and was therefore removed from further analyses (for analysis, including this individual, see Figure S1, Supplemental Material). It is hypothesized that this individual was significantly older than others in the study, including the other individual in the 45+ group. The older group contains the results of one individual.

### Analysis by Age

**Figure 2.**
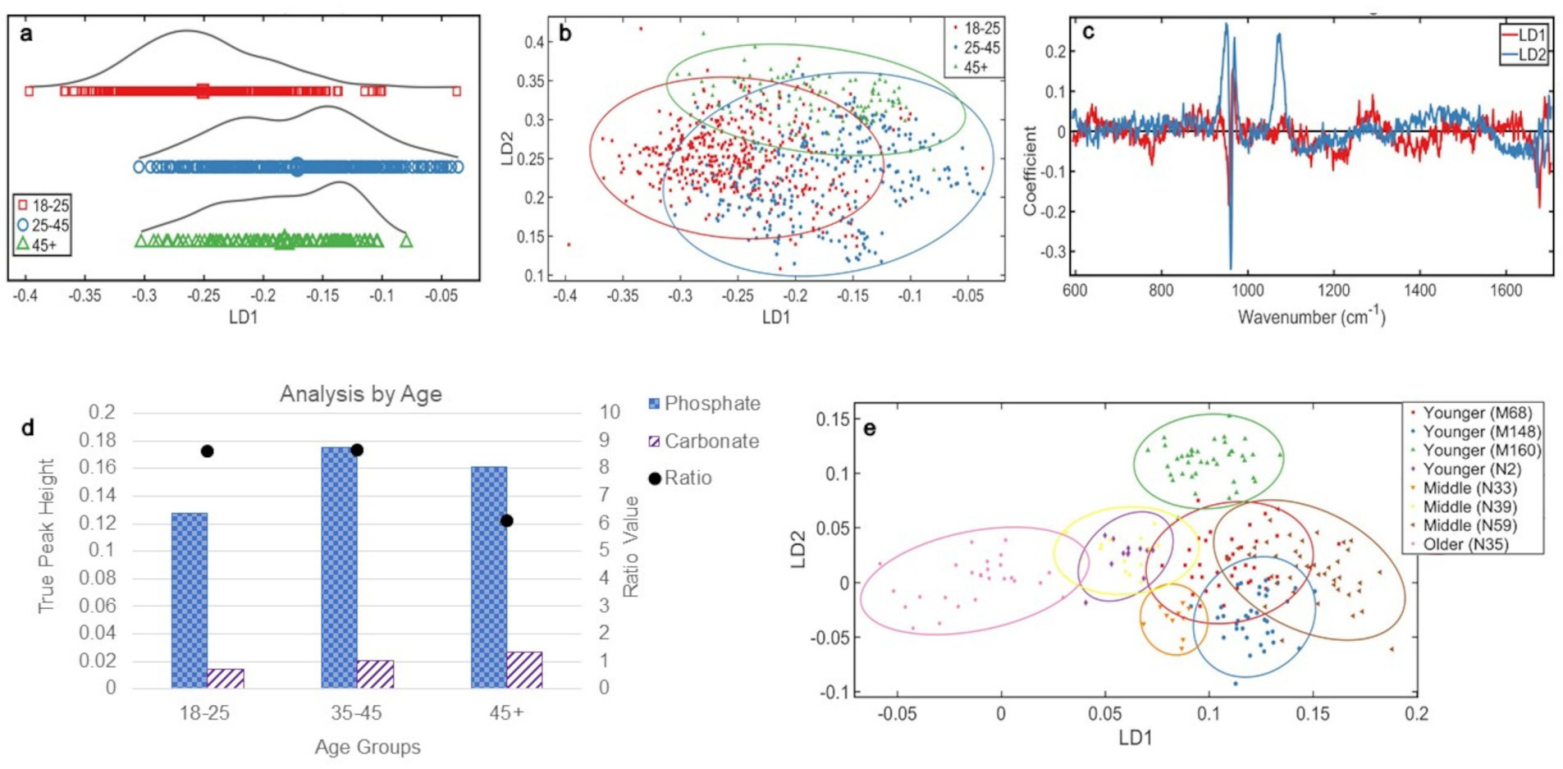
Analyses by age (a) 1D scores plot showing PCA-LDA. The averages of each class are indicated by an enlarged data point. (b) 2D scores plot of the three age groups. (c) PCA-LDA loadings for LD1 and LD2, illustrating all significant changes in bone chemistry with age. (d) Ratio plot illustrating the phosphate to carbonate ratio and the peak heights from which they are calculated. Analyses by individual L5 (e) 2D scores plot of the PCA-LDA of each individual in the study using only the L5 vertebrae. 95% confidence ellipses are displayed. The age class of each individual has been indicated.

The 1D scores plot (Figure 2a) showed substantial age group overlap. The younger group was normally distributed with a distinct average compared to the older groups that had similar averages to each other. The middle group was more heterogeneous, showing two distributions. Narrowing of the data with aging shows an increase in homogeneity.

Figure 2b showed an overlap of all age groups, but LD1 showed separation of the youngest group and LD2 showed separation of the oldest group.

Significant chemical changes with age responsible for these distributions were determined from the loadings (Figure 2c), in order of highest variance. Over LD1, these were phosphate (~960 cm^–1^) and amide I (∼1676 cm^–1^). Across LD2 were phosphate (∼960 cm^–1^) and carbonate (∼1074 cm^–1^). There was most carbonate in the oldest group, then the middle, and then the youngest, and there was most phosphate in the middle group, then oldest, and then youngest.

Overall, the combined amount of mineral increased with age (Figure 2d). However, while the amount of carbonate increased with age (36% increase from youngest to middle group, 31% increase from middle to older group, and 78% increase from youngest to oldest group), phosphate increased initially from the younger to the middle group (37% increase), but decreased from the middle to the older group (8% decrease), though this decrease still resulted in the older group containing more phosphate than the youngest (26% increase). The phosphate:carbonate illustrated these changes in mineral proportions, youngest to oldest: 8.6, 8.7, and 6.1. The considerable decrease in ratio for the older group reflects the decrease in phosphate, while the carbonate value continued to increase with age.

Mineral crystal maturity, determined by the width of the phosphate peak at half the height, showed the most mature crystals were in the middle group (14.3 cm^–1^), then the younger group (15.4 cm^–1^), and then the older group (17.3 cm^–1^).

### Analysis of the Fifth Lumbar Vertebrae

While an age range for each individual was assigned, the historical and incomplete nature of each individual's skeleton makes precision difficult. Analyses by individual and age using only L5 vertebrae displayed a different distribution of the data than when including all vertebrae.

Analysis of each individual's L5 vertebra (Figure 2e) demonstrated distinction of the oldest individual. There was full separation of the three individuals of the middle group from each other, but a reasonable homogeneity was observed as distribution of these groups is tightest. This was similar for the younger group, with the exception of one individual (M160), which was distinct from all other individuals (Figure 2e).

**Figure 3.**
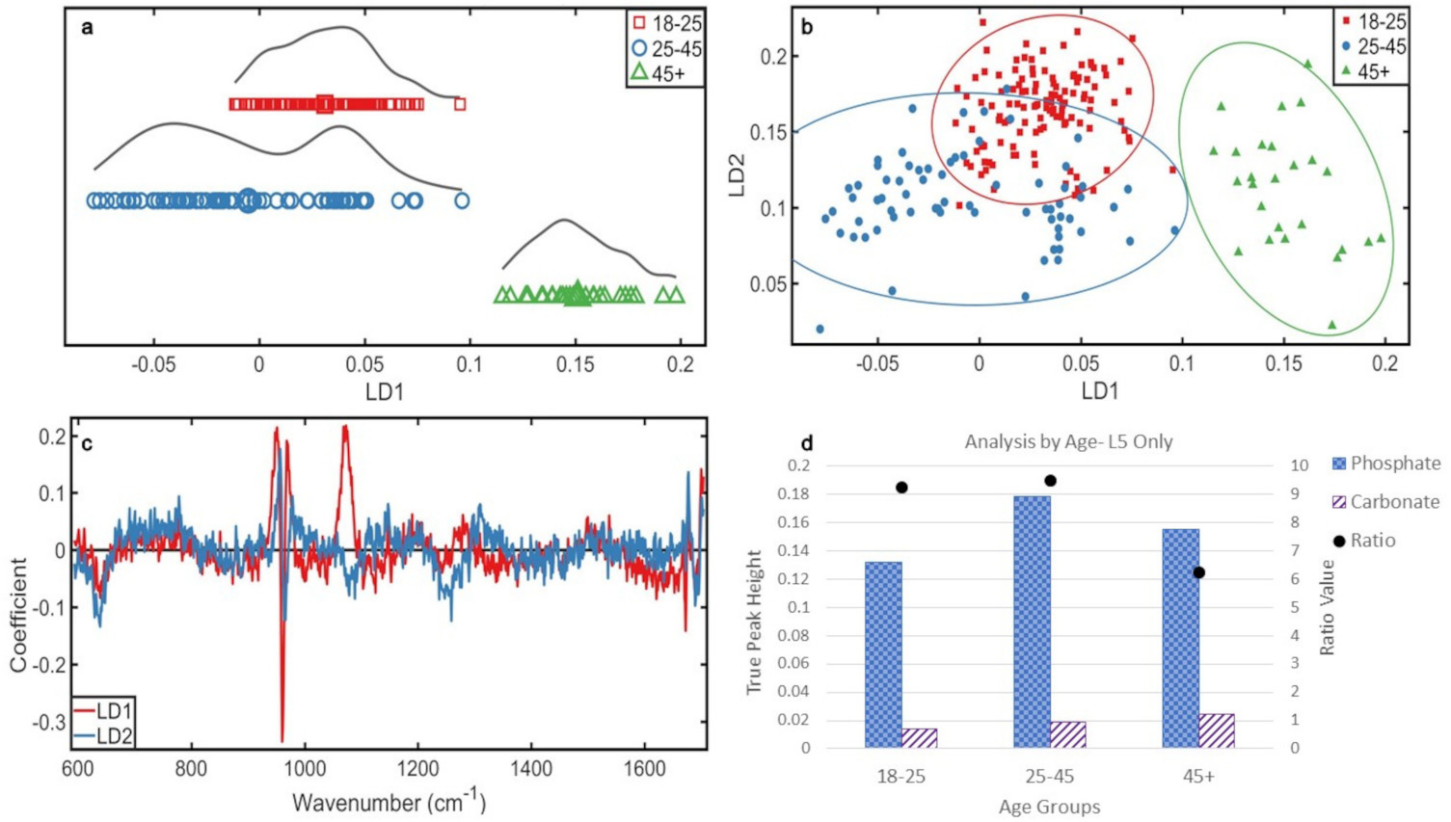
Analyses by age, L5 only: (a) 1D scores plot showing PCA-LDA of L5 data classed by age. The averages of each class are indicated by an enlarged data point. (b) 2D scores plot of the three age groups using the L5 data. (c) PCA-LDA loadings by age in the L5 vertebrae. (d) Ratio plot illustrating the phosphate to carbonate ratio and the peak heights from which they are calculated.

The PCA-LDA scores plot (Figure 3a) of the L5 vertebra by age showed normal distribution of the youngest and oldest group, with the middle group demonstrating a (biphasic) split. The first peak in the middle group was distinct from the second, which overlapped with the younger group. The older group was distinct from the other groups (Figure 3a).

In the 2D scores plot (Figure 3b), there was overlap between the middle and youngest (most homogeneous) group; the older group was distinct, which is significant as demonstrated by 95% confidence intervals.

The chemical changes (Figure 3c) responsible, in order of highest variance, for these distributions were phosphate (∼960 cm^–1^), carbonate (∼1070 cm^–1^), and amide I (∼1672 cm^–1^) over LD1 and phosphate (∼960 cm^–1^) and amide I (∼1677 cm^–1^) over LD2.

Most carbonate was present in the oldest group, then middle, and then youngest group. The middle group contained the most phosphate, then the oldest, and the youngest. The younger and middle groups were more similar over LD1 than LD2 (Figure 3b), and from the loadings and ratios (Figure 3d), it is likely that this stronger LD2 change is a result of phosphate increase from ∼25 to ∼45 years.

Overall, mineral increased with age in L5 (Figure 3d). Carbonate increased with age (32% youngest to middle, 32% middle to oldest, 74% youngest to oldest), and phosphate increased initially from youngest to middle group (35% increase), but decreased from the middle to the older group (13% decrease), though this decrease still resulted in the older group containing more phosphate than the youngest (17% increase). The phosphate:carbonate, from youngest to oldest, was 9.2, 9.5, and 6.2. The considerable decrease in ratio for the older group reflects the decrease in phosphate, while the carbonate value continued to increase with age.

The most mature mineral was present in the middle group (13.9 cm^–1^), followed by the youngest group (15.21 cm^–1^) and then the oldest group (17.98 cm^–1^).

To investigate if there were differences in bone chemistry between joint type, the Z1 and Z2 measurements (Figure 1e) were separated from the V1 measurements (Figure 1d) of the L5 vertebrae.

**Figure 4.**
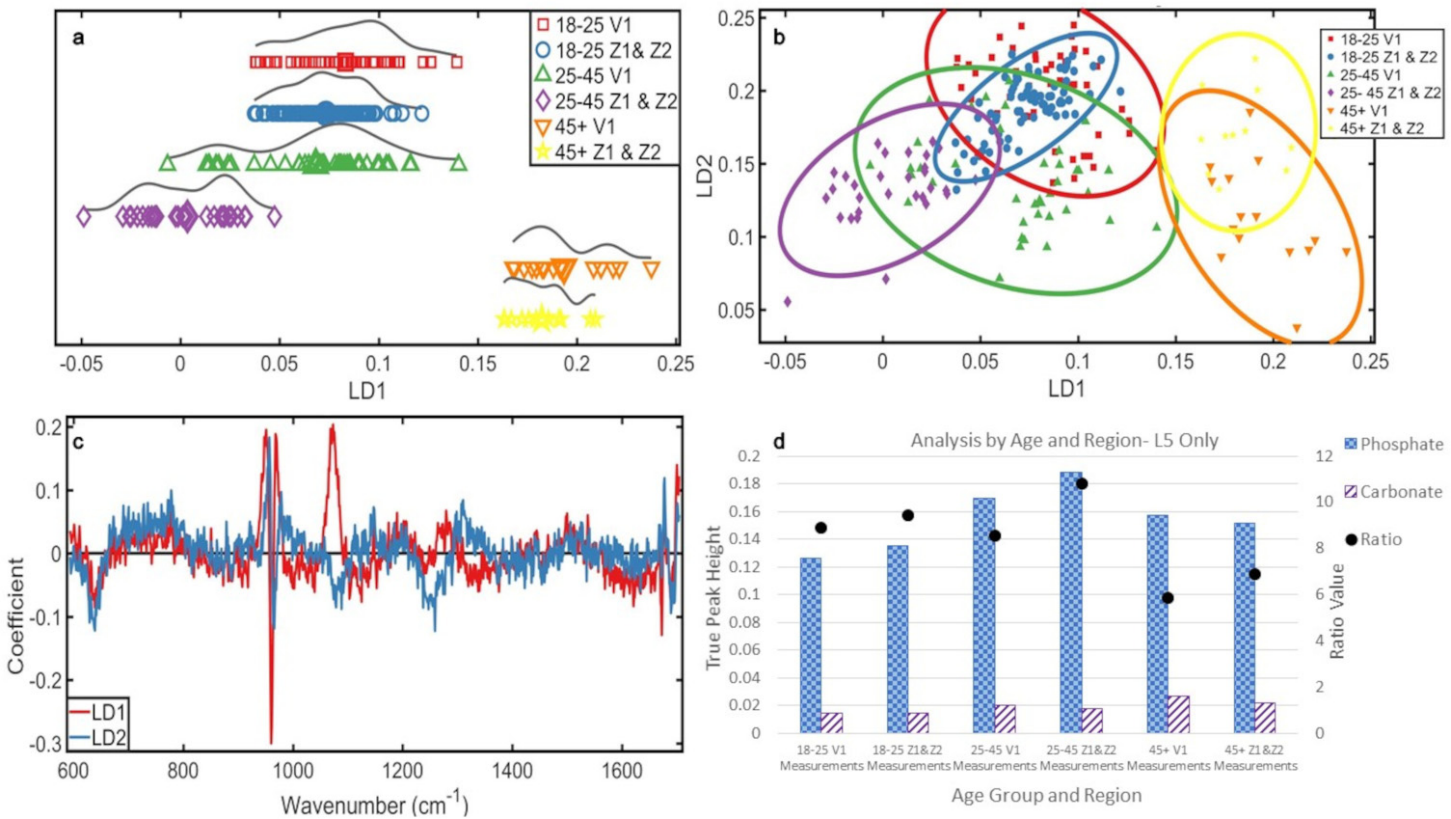
Analyses by age and grouped region L5 only: (a) 1D scores plot showing PCA-LDA of L5 data classed by age and grouped region. The averages of each class are indicated by an enlarged data point. (b) 2D scores plot of the age groups using the grouped region L5 data. (c) PCA-LDA loadings of age and grouped region in the L5 vertebrae. (d) Ratio plot illustrating the phosphate to carbonate ratio and the peak heights from which they are calculated.

The LD1 scores plot (Figure 4a) showed the L5 s of the younger group possessed similar chemistry between regions and the Z measurements lay within the average of the V measurements. However, the middle group showed a difference in chemistry between grouped regions; the V measurements overlapped with the younger group, though with less homogeneity, and the Z measurements showed a split in the data, the distribution overall placed them further left of the LD1 axis than the younger measurements. The older group L5 regions showed similar distribution and spread and overlapped considerably, but were distinct from other age groups (Figure 4a).

When LD2 distribution is considered (Figure 4b) the Z measurements for the older group L5 s aligned with both of the youngest measurements, the V measurements were more aligned with the middle group measurements.

The chemical changes responsible for these distributions (Figure 4c) were (in order of highest variance) phosphate (∼960 cm^–1^) and carbonate (∼1070 cm^–1^) over LD1 and phosphate (∼960 cm^–1^) over LD2. Phosphate quantity was highest in the middle group, Z then V measurements. The oldest group had the next highest quantity of phosphate, but the V measurements contained more phosphate than the Z measurements. The youngest group contained the least phosphate and the Z measurements contained more than the V measurements (Figure 4d). Phosphate therefore followed the same pattern with aging as analyses before being split into measured regions, regardless of measurement location. However, the Z measurements contained more phosphate than the V measurements in the younger and middle groups. This was reversed in the older group.

Carbonate was highest in the V measurements of the oldest group, then the Z measurements. Both the Z and V measurements of the middle and younger groups were similar in their intensities. This indicates that while there may be a change in carbonate intensity of the V and Z measurements before ∼45 years within a vertebra, they are not strong enough to be detected with this sample size.

Overall mineral increases with age in L5 and carbonate increase mirrors this while there is a reduction in phosphate between the middle and older groups that causes the ratio of these minerals to change with aging. When analyzed by measured region, there was a consistently higher phosphate:carbonate in the Z measurements than the V measurements in each age group (Figure 4d). Due to the proximity of the detected carbonate intensities in the younger and middle group's isolated V and Z measurements, this relationship cannot be certain within the scope of this study. However, the older group's carbonate measurements were distinct and showed the V measurements were higher than the Z measurements (V 22% higher than Z).

Phosphate was higher in the Z measurements compared to the V measurements for the younger (Z 8% higher than V) and middle group (Z 11% higher than V), but was higher in the V measurements than the Z for the oldest group (Z 6% lower). The phosphate increased from the younger to the middle group, but decreased from middle to older group, maintaining an increase with age overall, despite separation of measurements.

The most mature mineral was present in the middle group, with both V and Z measurements presenting equal maturity (14.14 cm^–1^). The youngest group contained the next most mature crystals: Z measurements (15.20 cm^–1^) and then V measurements (15.22 cm^–1^). The least mature mineral was in the older group, with V measurements (17.20 cm^–1^) more mature than Z (18.14 cm^–1^).

Due to the nature of archaeological material, the zygapophyseal joint facets were not always present, meaning there was inconsistency in obtaining the Z1 and Z2 measurements across samples. To eliminate this variable, and its influence on the chemical trends observed, the V measurements of the L5 vertebrae were isolated and analyzed.

**Figure 5.**
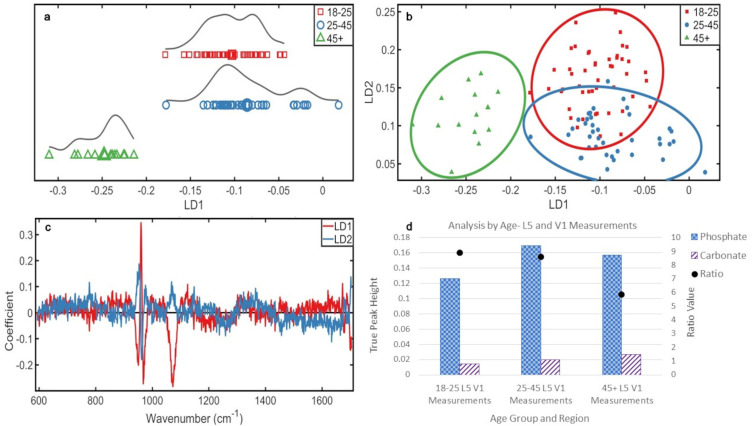
Analyses by age, L5 V1 measurements only: (a) 1D scores plot showing PCA-LDA of V1 measurements from L5 vertebrae. The averages of each class are indicated by an enlarged data point. (b) 2D scores plot of the three age groups using the V1 data from L5 vertebrae. (c) PCA-LDA loadings of age in the V1 measurements of the L5 vertebrae. (d) Ratio plot illustrating the phosphate to carbonate ratio and the peak heights from which they are calculated.

Figure 5a showed that all three groups had split distributions, younger and middle groups overlapped, the older group was distinct and more homogeneous.

In the 2D scores plot (Figure 5b), there was separation of the younger and middle groups over LD2, but overlap was still present. LD1 separates the older group.

The chemistry responsible for these distributions (Figure 5c) were (in order of highest variance) phosphate (∼960 cm^–1^) and carbonate (∼1070 cm^–1^) over LD1 and phosphate (∼960 cm^–1^) over LD2. Most phosphate was present in the middle group, then oldest group, and then youngest group. Most carbonate was in the oldest group, then the middle group, and then the youngest (Figure 5d).

Mineral crystals were the most mature in the middle group (14.14 cm^–1^), followed by the youngest group (15.22 cm^–1^), and then the older group (17.20 cm^–1^), which is a pattern that was consistent with all previous analyses.

The amount of carbonate increased with age (40% youngest to middle, 35% middle to oldest, 89% increase youngest to oldest) and phosphate increased with age initially from youngest to middle (35% increase), but decreased from middle to oldest group (7.5% decrease). However, phosphate still increased with age between the youngest and oldest group (24%). Despite the patterns of increase and decrease being consistent with other analyses, the ratios did not mirror this.

In all other analyses, phosphate:carbonate increased between younger and middle groups and decreased between middle and older groups due to the decrease in phosphate with the continuing rise in carbonate between the middle and older groups. When the L5 V measurements were isolated, however, the ratio decrease in the older group remained, but the middle group more closely mirrored the youngest. From youngest to oldest, these ratios were 8.9, 8.6, and 5.7, indicating that the carbonate rise in the middle group was higher than the phosphate increase, which was not detected in earlier analyses.

### Analysis of the Vertebral Body Measurements by Vertebrae and Age

To further scrutinize if the differences observed in these V measurements were unique to L5 or a consistent pattern, the V measurements of L3, L4, and L5 were compared.

**Figure 6.**
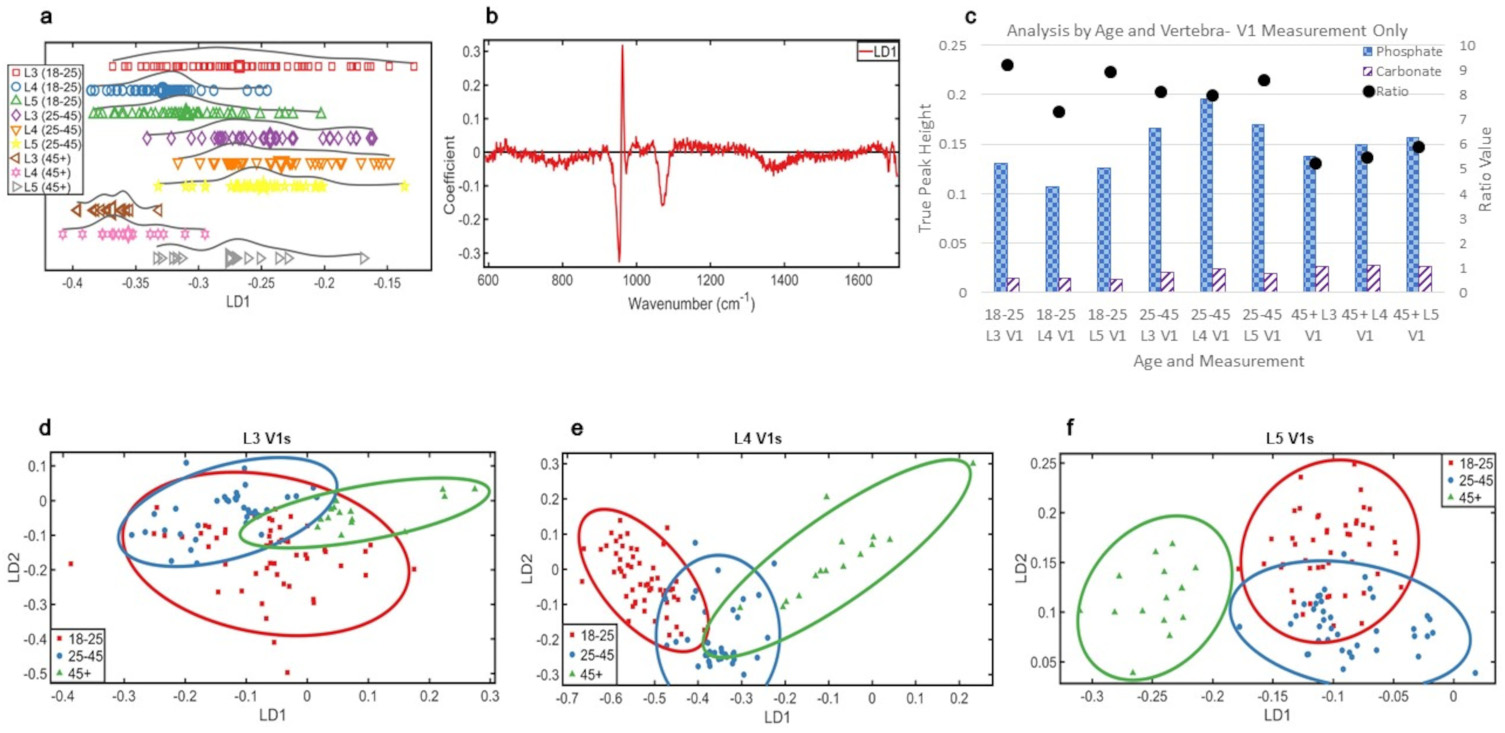
Analyses by vertebrae and age, V1 measurements only: (a) 1D scores plot showing PCA-LDA of all V1 measurements classed by age. The averages of each class are indicated by an enlarged data point. (b) PCA-LDA loadings of age in the V1 measurements. (c) Ratio plot illustrating the phosphate to carbonate ratio and the peak heights from which they are calculated. (d) 2D scores plot of the three age groups using the V1 data for L3. (e) 2D scores plot of the three age groups using the V1 data for L4. (f) 2D scores plot of the three age groups using the V1 data for L5.

A 1D scores plot (Figure 6a) showed the youngest L3 s were the most heterogeneous. All three vertebrae of the middle group had similar distribution, and the oldest group demonstrated an increasing heterogeneity from L3 to L5, with a difference in the L5 s compared to the L3 and L4 of the same age group. There was a decrease in homogeneity over L3 with age that also existed within L4, but was less defined.

The comparisons of the 2D scores plots (Figure 6d–6f) showed the mineral chemistry of the older group became more distinct the lower down the spine the data were collected. There was consistent overlap of the younger and middle groups within each vertebra, which was most pronounced in L3.

The loading chemistry responsible for the changes in Figure 6a is shown in Figure 6b. These were phosphate (∼960 cm^–1^) and carbonate (∼1070 cm^–1^) over LD1. Over LD2, the corresponding loadings for Figures 6d–6f (cf. Figure S2, Supplemental Material) illustrated that only phosphate (∼960 cm^–1^) was responsible for mineral changes in the L3, L4, and L5 vertebrae.

The middle group contained the most phosphate overall; L4 contained the most, then L5, and then L3. The L5 and then L4 of the oldest group had the next highest intensity, but the younger L3 measurements had a higher intensity than the older group's L3. The youngest group L5, then L4, had the lowest phosphate intensity. Most carbonate was in the oldest group, and all three vertebrae had similar intensity. Similar to analyses by measured region, the middle and younger groups overlapped considerably over the carbonate peak.

The phosphate:carbonate (Figure 6c) had a trend of decreasing with age in L3 (9.2, 8.1, and 5.2) and L5 (8.9, 8.6, and 5.9), but increased from younger to middle group in L4 before decreasing between middle and older group (7.3, 7.9, and 5.5). The oldest group always had the lowest ratio regardless of vertebral level. While this indicates potential changes within the spine between lumbar levels, it can only be considered as a trend.

The most mature crystals were in middle group: L4 s (14 cm^–1^), L5 s (14.1 cm^–1^), and then L3 s (14.2 cm^–1^). The youngest L3 s (14.5 cm^–1^) and L5 s (15.2 cm^–1^) contained the next most mature mineral, but the older L5 s (17.2 cm^–1^) were of similar maturity to the youngest L4 s (17.5 cm^–1^). The least mature mineral was in the older group: L4 s (17.7 cm^–1^) and then L3 s (18 cm^–1^).

## Discussion

This study is the first to analyze the historically significant bone samples from Thornton Abbey using Raman spectroscopy. The aim was to determine if changes in bone mineral chemistry with age could be detected in human archaeological lumbar vertebrae. The data were analyzed by age, by vertebra, and by the measured region, which were selected for their biomechanical significance (Figure 1c).

Phosphate is a substantial component of bone mineral (hydroxyapatite) and demonstrated the strongest peak in these samples at ∼960 cm^–1^ (Figure 1f). The second most prominent peak was carbonate ∼1070 cm^–1^ (Figure 1f), an influential integrated component of skeletal apatite.^23^ Bone mineralization is fundamental to bone quality,^24^ with an increase in mineral components like phosphate with age, as this study has found, increases the stiffness of bone, compromising flexibility and affecting function. When a biomechanical threshold is reached, this can elevate to conditions like osteoarthritis, where phosphate is the primary bone mineral affected,^25,26^ and the change in bone structure yields a breakdown of joint surfaces, or osteoporosis, where reduced bone mineral density and increased mineralization result in weaker bones with higher fracture risk.

While inorganic mineral was intact in these samples, the organic collagenous peaks found in fresh bone were not consistently present because of poor and variable preservation of proteins in archaeological remains.^12,21^

Age-related changes in the skeleton in twentieth- and twenty-first-century populations are reasonably well understood at a gross level. However, the samples in this study date to the fourteenth century during the Black Death when life expectancy was ∼25 years.^27^ Although this figure is skewed by particularly high rates of infant and child mortality, for those reaching adulthood, prospects would have improved significantly. Nonetheless, this is still lower than the preceding and subsequent centuries and significantly shorter than the current UK life expectancy.^28^

The estimated 48 individuals interred at Thornton Abbey are thought to be part of the local secular community, due to the presence of men, women, and children as young as one year-old, rather than the exclusive population of the monastery.^2^ As this community would be from a more rural than urban setting, with a lower population density and unintentionally better sanitation, it might be presumed that their average life expectancy could be slightly higher and so there could be more variation in the older group than if these remains were from an urban population.^27,29^ However, those living in rural environments were still frequently subjected to periods of famine, disease, and more arduous physical labor than their urban counterparts. Given this, these historical environmental stresses do not directly transfer to the modern population, so must be considered within the context of these results.

The samples in this study represent adults across a range of ages (18–45+). Their ages were estimated using dental and skeletal growth and development indicators in non-adults, with degenerative skeletal changes and dental information for adults.^2^ These methodologies are considered accurate in determining approximate age. However, biological variation can result in overlap in age and biological processes with adjoining groups.^30^ Individuals estimated to be over 45 years were combined into a group due to the increased difficulty of skeletal age determination after this approximate age.^31^ There is a wide age range within these groups: one individual could be considerably younger than another, e.g., in the middle group, one individual could have been 26 at the time of death and another 40. Overlaps in the scores plots are likely attributed to this.

The L3–L5 analysis by age gives an overview of lower lumbar spine health; an area of modern importance and historical interest as it carries the greatest load during life and will likely be the location of representative changes.

From its chemistry, one of the samples is estimated to be significantly older than its counterpart in the same age group (45+). Analysis inclusive of this sample can be found in Figure S1, Supplemental Material. This sample was so significantly different that it masked differences between and within age groups. These considerable changes in the mineralization may also be associated with age-related bone disease, such as osteoarthritis.^14,32^

### Analyses by Age

Although individuals’ bones will not age the same,^33^ combining several of similar ages is the best approach for an overall picture. Overall, mineralization increased with aging (Figure 2) and the age range nature of the samples allowed transitional chemical changes to be observed (Figure 2a and 2b). The data shows individuals at the higher or lower end of their determined age bracket aligned in compositional proximity with individuals at the end of the bracket next to theirs. There is more chemical heterogeneity in younger lumbar vertebrae than in older, and there is certainty that individuals in these groups would not overlap in biological age.

Carbonate increased with age. The middle group has the most phosphate, decreasing to the older group. The youngest group possessed the lowest amount of both minerals. These patterns in mineral change with age show that the two most abundant minerals in bone^8,33^ increase in age, but at ∼45 years, phosphate begins to decrease while carbonate continues to rise. Despite the phosphate decrease, there is an overall rise in mineral quantity, indicating a greater increase in carbonate than a decrease in phosphate (Figure 2d). The effects of these two findings could be instrumental to our understanding of bone mineral aging. An overall increase in mineral suggests lumbar vertebrae are becoming more brittle with age, as previously described.^33^ However, this should not be considered in isolation as the combined effect of an increase in carbonate with a decrease in phosphate impacts skeletal function differently than an overall increase in mineral.

Mineral maturity is also important; the middle group possessed the most mature mineral and the oldest group the least mature. This is consistent with studies that demonstrated an increase in maturity during the first ∼30 years of life, but a decrease afterwards;^33,34^ therefore, 25–45 is the predicted age of peak bone mass/stabilization. The least mature crystals being in the oldest group suggests the adult skeleton has larger crystals before this apex of crystal size at ∼30 years than is present in older skeletons. This could be from decreased bone remodeling with age as bone turnover slows and the amount of bone deposited decreases.^35^

### Fifth Lumbar Vertebra

Vertebra L5 receives the greatest compressive load in the human spine;^36^as such, it may show the earliest chemical variances from aging or pathologies making it a useful resource about health and disease and a current area of assessment in NHS procedures examining bone health.^15^ Loading bones is important to stimulate bone growth and maintain a healthy bone environment; mechanical loading causes new bone deposition in regions of high stress, producing the most efficient mechanical structure.^37^

Figure 2e shows the middle group are the most distinct from each other, potentially demonstrating that age-related mineralization changes start to happen at 25–45 before becoming a distinct chemical change in the older group. One individual in the middle group (N59) is more aligned with the younger individuals (18–25 years), suggesting they are on the younger end of the middle age range. Furthermore, one younger individual (N2) is much closer to the middle and older individuals, suggesting they are at the higher end of their age range, or may have pathologies prematurely adjusting the skeletal age.

The L5 s had more pronounced changes with age; each group was more homogeneous and distinct (Figure 3a and 3b). The oldest group did not overlap the other age groups, showing that changes with age in L5 after ∼45 years are significantly more pronounced than changes in the L5 of younger spines. Carbonate increased in individuals ∼45+ causing this distinction, which is more pronounced in L5.

The phosphate:carbonate in L5 suggests an overall higher mineralization than more superior lumbar vertebrae, but this is not significant.

The lifestyle of these individuals from a medieval, rural community could also result in changes in bone chemistry, e.g., hard manual labor causing premature skeletal aging, particularly in the lower spine.^38,39^ Consequently, some unmeasurable variables could be contributing.

### Regional Analyses

The shape of the lower lumbar vertebral bodies distort with age and disease: their height decreases, and width increases due to compression from the trunk over life. This morphological change results in alterations to spinal biomechanics.^40^ Furthermore, biomechanical adjustments result in altered bone remodeling and could contribute to pathologies and fracture risk. The zygapophyseal joints are more subject to the direct forces of muscles and ligaments; these forces also contribute to the locations of osteoarthritis occurrence, but their biomechanical contribution receives different force distribution than those on the vertebral body. As such, these measurement areas were separated to investigate if differences in bone mineral chemistry could be detected and potentially attributed to differences in force dynamics.

There was no significant difference related to aging associated with L5 analyzed by region (Figure 4a and 4b). However, the incomplete nature of archaeological material could cause inconsistency in measurement distribution within age groups (discrepancies in the presence of V and Z measurement locations can be found in Table S1, Supplemental Material). The younger group had no significant variance between their V and Z measurements, suggesting mineral changes are more consistent across the whole bone, potentially as biomechanical stress factors are less influential in young adults, even if they were involved in heavy manual labor in a rural medieval community.^41^

The middle group has differences between the V and Z regions, with the former presenting similar chemistry to the younger group's regions (Figure 4a and 4b). This suggests that when changes in bone chemistry occur with aging, they are more likely present in the Z-joint facets than at the vertebral end plates. This is potentially due to the smaller surface area at the Z joints compared to the size of the vertebral body. However, that would be dependent upon higher stress factors in conjunction with the aging skeleton, which are an unknown variable. The older group present similarities in their regions of measurement, but are distinct from other age groups, indicating the location of changes balance out again over the whole bone by ∼45 years. The changes were significantly associated with phosphate and carbonate (Figure 4c), indicating mineral changes, and the separation of the older age group V measurements being so different, compared to the more similar younger and middle age group's V measurements, appears to be a result of a sharp rise in carbonate (Figure 4d) with the carbonate rise in the Z measurements in the older age group being less pronounced. This imbalance in rising carbonate levels between the V and Z measurements of the older age group could be indicative of the vertebral body showing a more sudden change as a result of a biomechanical threshold for the lower spine being reached, and the Z measurements, being less effected by the upper body weight, display a more gradual adjustment.

The phosphate:carbonate was consistently higher in the Z measurements with aging (Figure 4d), and similar to phosphate and carbonate quantity in previous analyses, however, the distribution of the minerals between measurement sites differed. Phosphate was higher in the Z measurements of the younger and middle groups than the corresponding V measurements, but in the older group, phosphate was higher in the V measurements than the Z. This could suggest higher mineralization in the vertebral body than the Z joints in individuals ~45+ due to less mobility at the Z joints with aging, or the consistent weight bearing of the L5 vertebral body in life requiring a significant compositional adjustment in the bone mineral to adapt with aging. In contrast, the carbonate content was higher in the V measurements in the middle and older groups, but was higher in the Z measurements of the youngest group. This could indicate that while there is an overall increase in mineralization with aging, the higher carbonate in the Z joints of younger individuals could be linked to the increase in bone remodeling from activity, as the Z joint forces are more linked to stability in movement and muscle forces. The higher carbonate content in the vertebral bodies of the middle and older groups could be a result of the bone mineral increasing from increase in load demands with aging from reduced movement. This uneven mineral distribution through the vertebra highlights that potential changes could be associated with biomechanical loading and movement.

The V measurements were present on all samples (as shown by the measurement location presence breakdown in Table S1, Supplemental Material) and are important in relation to potential osteoporotic and osteoarthritic chemical changes, particularly in L5 due to its location as the final force transfer point from trunk to pelvis.

There are similarities in the younger and middle groups, and the older group is distinct (Figure 5b). This indicates that while a gradual change in bone mineral chemistry with age was detected, these changes occur at an accelerated rate with increased age. It is assumed that samples from the younger group that do not overlap with those from the middle group are the youngest individuals from the dataset; those that overlap are more similar in age of late 20s to early 30s. The older group demonstrated a greater degree of homogeneity, but this group only contains one individual's measurements. The chemical causes of this distribution were phosphate and then carbonate (Figure 5c), with more variance in both minerals in the older group compared to the middle and younger group V measurements, which is consistent with previous analysis of L5 vertebrae with all measurements included. The phosphate and carbonate quantity are also consistent with previous analyses (Figure 5d), with an increased mineral quantity with age and overall rise in carbonate and an initial rise in phosphate, which decreases between the middle and older groups. However, the ratio pattern for isolated V measurements shows the first deviation from the previous results. In earlier analyses, the phosphate:carbonate increased from the younger to middle group and then decreased between the middle and older group as a result of the decrease in phosphate with the continuous rise in carbonate with aging. Here, the decreasing phosphate:carbonate with age indicates that while there was more phosphate present in every age group, carbonate increase was higher than phosphate increase in the middle group. This pattern is exclusive to the V measurements of L5. An increase in carbonate could affect cellular activities and bone health, as carbonate increases the solubility of the hydroxyapatite in bone, prompting bone turnover.^33^

The most mature mineral was in the middle group, then youngest, and then oldest. This could be attributed to the middle age range having a period of mineral stability with reduced turnover so mineral is present for longer.^33,37^ The youngest group is next due to the high bone turnover in the younger skeleton, and the least mature detected in the older group due to age-related changes causing altered mineral content. Though the differences are slight, they are still present. The amount of these significant minerals did increase with age, but not proportionally, as phosphate increased more with age than carbonate.

Overall, chemical variance decreases with age across all three vertebrae when using the V measurement (Figure 6a). However, the significance decreases from L3 to L5, with the middle group appearing the most similar in their chemistry within the spine. This shows that the younger vertebrae are more diverse in their bone chemistry and signs of this decreased variance begin to show in L3 before descending to L4 and L5. Considering the biomechanical importance of these bones, this could be compensatory changes being detected in the higher vertebrae.

The separation of the oldest group becomes more pronounced further down the spine. These are predominantly from changes in phosphate and, less so, but still significantly, carbonate, effectively showing changes in bone mineral with age. The data supports the idea that there are stronger changes in phosphate at the V measurements between all three vertebrae than when all measurements were combined or L5 in isolation.

The maturity of the mineral crystals aligns with earlier results that the middle group possesses the most mature mineral, but with no discernible pattern within the spine. The youngest and older groups overlapped in their maturity when the V measurements were considered by vertebrae. This suggests that this study's earlier assumption that the increase in crystal maturity during the first ∼30 years yields more mature crystals than those in individuals ∼45+ years is valid, when all measurements are combined by age. However, it does not hold true for vertebrae when investigated individually. This overlap indicates the middle group consistently has the most mature mineral, but the increase in the adult skeleton up to ∼30 years to get to this point is not consistently higher than the decrease in mineral maturity found in the ∼45+ group. The L4 of the younger and middle groups has the lowest mineral ratio of the three measured vertebrae, with the L3 and L5 ratios being higher. This was not observed in the oldest group where the mineral ratio was found to increase from L3 to L5. This finding could also be linked to compensatory changes of the L4 vertebrae in the younger and middle group given the likely biomechanically stressful lifestyle in rural medieval England. This differing pattern in the older group could be a result of the compensatory changes reaching a biomechanical threshold, where the bone chemistry evens out to best adapt to the skeletal demands in an older skeleton.

## Conclusion

Raman spectroscopy detected significant age-related changes in bone mineral chemistry, associated with changes in phosphate (∼960 cm^–1^) and carbonate (∼1070 cm^–1^). The phosphate peak contains the most information across all analyses. Amide bands were detected in these analyses, but their relationship to changes with aging was not investigated due to the inconsistent preservation of protein in archaeological bone making protein measurements unreliable.

The L3–L5 lumbar vertebrae and their regions were selected for their biomechanical importance. Specifically, the V measurements are from the vertebral body which forms the column of the spine and receives the highest loading in life in both passive and active movements. The Z regions are taken from the zygapophyseal joints which are integral in structural stability during spinal movement. Changes with age were found in all three vertebrae at both the V1 and Z1 and Z2 regions. However, they were stronger in the L5 vertebrae, with an indication that the vertebral body measurements contain the most information regarding these bone mineral chemistry changes. These age-related changes were associated with variance in phosphate and carbonate. With aging, the amount of carbonate increases, but phosphate only increases up to ∼45 years and then decreases. Ratios showed this phosphate decrease was less than the carbonate increase for all analyses, resulting in an overall increase in mineral with age. In addition, when the V measurements of the L3 and L5 were investigated, the middle group had a proportionately higher rise in carbonate than in phosphate, leading to an overall rise in ratio with age.

There was a consistently higher phosphate:carbonate in the Z measurements. However, mineralization was higher in the V measurements than the Z in the oldest group only due to the inconsistency in zygapophyseal joint presence, requiring further study.

This study has added to the body of work relating to individuals who lived and died at Thornton Abbey, but these historical remains have also contributed to modern understanding of changes in bone mineral chemistry with age in the human lumbar spine. Changes in bone chemistry can be detected with Raman spectroscopy, and detailed fluctuations in bone mineral chemistry could provide valuable insights into causes, and therefore treatments, of mineralization-related diseases.

## Research Limitations

Access to human archaeological remains is easier than modern samples, but the nature of archaeological material limits usable samples from an assemblage, which in turn restricts sample size. These samples were the most intact and best preserved from the 21 adult human skeletons,^2^ but there was still a lack of consistency in measurable areas due to degradation, which was a limitation.

## Supplemental Material

sj-docx-1-asp-10.1177_00037028241291601 - Supplemental material for Raman Spectroscopy Detects Bone Mineral Changes with Aging in Archaeological Human Lumbar Vertebrae from Thornton Abbey Supplemental material, sj-docx-1-asp-10.1177_00037028241291601 for Raman Spectroscopy Detects Bone Mineral Changes with Aging in Archaeological Human Lumbar Vertebrae from Thornton Abbey by Sheona Isobel Shankland, Hugh Willmott, Adam Michael Taylor and Jemma Gillian Kerns in Applied Spectroscopy
