# Supplementary material for: Raman Spectroscopy Detects Bone Mineral Changes with Aging in Archaeological Human Lumbar Vertebrae from Thornton Abbey
Source: Appl Spectrosc. 2024 Nov 8;79(3):413–25. doi: 10.1177/00037028241291601 (PMC11898377; doi:10.1177/00037028241291601)
Supplement: sj-docx-1-asp-10.1177_00037028241291601 - Supplemental material for Raman Spectroscopy Detects Bone Mineral Changes with Aging in Archaeological Human Lumbar Vertebrae from Thornton Abbey [file sj-docx-1-asp-10.1177_00037028241291601.docx]

**Supplemental Material**

**Raman Spectroscopy Detects Bone Mineral Changes with Aging in Archaeological Human Lumbar Vertebrae from Thornton Abbey**

Sheona Isobel Shankland*, Hugh Willmott, Adam Michael Taylor, and Jemma Gillian Kerns

Lancaster Medical School, Lancaster University, Lancaster, UK LA1 4YG

*Corresponding author email: Sheona Isobel Shankland: s.shankland@lancaster.ac.uk


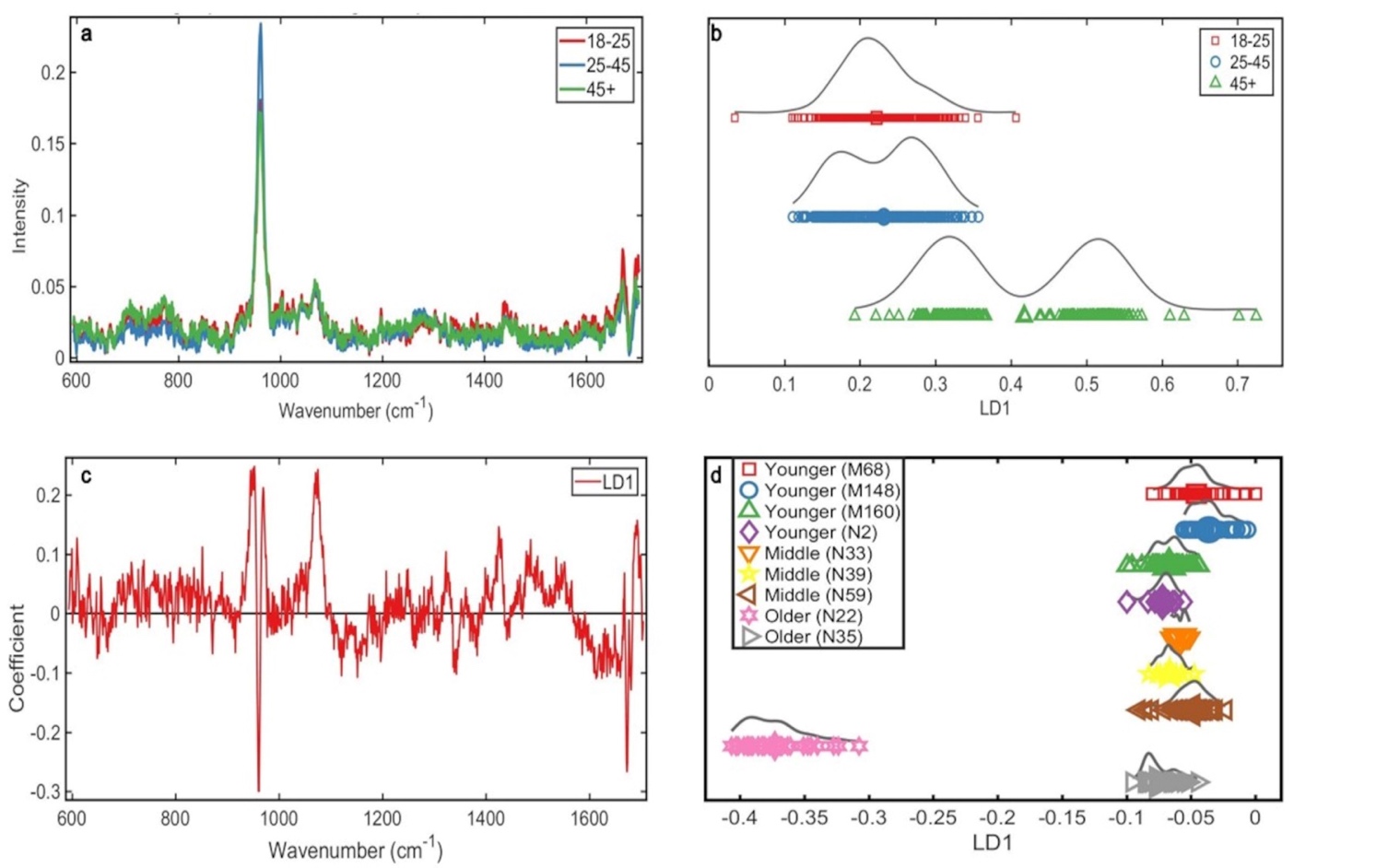


**Figure S1.** (a) Average spectra of all measurements from all vertebrae classed by age. (b) 1D scores plot showing PCA-LDA of all measurements from all vertebrae classed by age. The averages of each class are indicated by an enlarged data point (c) PCA-LDA loadings for LD1, illustrating all significant changes in bone chemistry with age across all vertebrae in the study. (d) 1D scores plot of each individual in the study illustrating the significant chemical changes detected in individual N22 masking any other changes present, thus justifying its removal from this study.


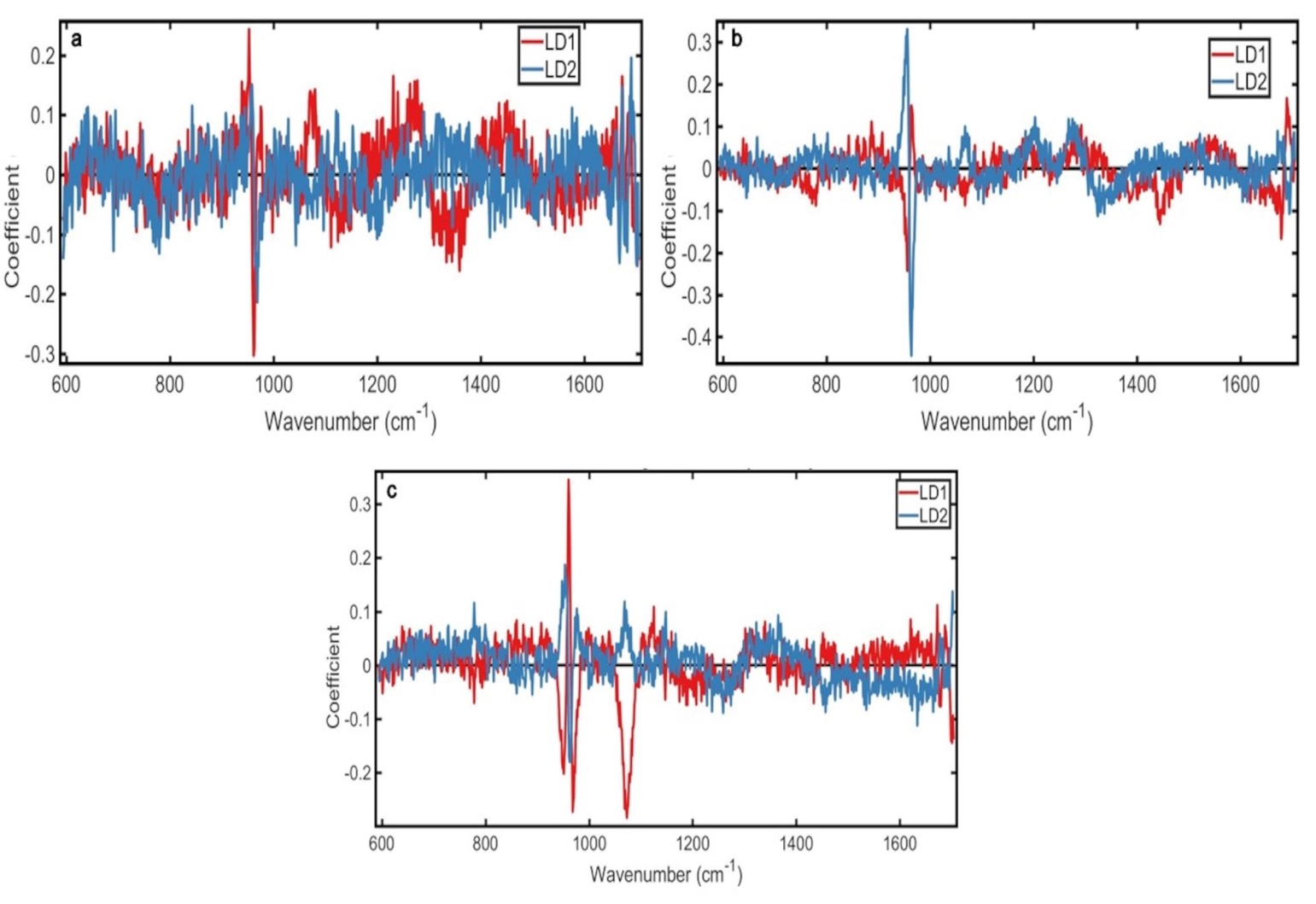


**Figure S2.** (a) Loadings for PCA-LDA analyses on L3 vertebrae V1 measurements when classed by age. (b) Loadings for PCA-LDA analyses on L4 vertebrae V1 measurements when classed by age. (c) Loadings for PCA-LDA analyses on L3 vertebrae V1 measurements when classed by age.

**Table S1.** Sample list stating age group and skeletal contents of each individual. The presence of each of the three measurable areas on each vertebra has been indicated as well as the number of spectral measurements taken for each area, if present.

| Skeleton number | Estimated age | Vertebrae | Area of measurement | Number of spectra |
| --- | --- | --- | --- | --- |
| N002 | 18–25 years | L3 | V1 | 12 |
|  |  |  | Z1 | 12 |
|  |  |  | Z2 | 15 |
|  |  | L4 | V1 | 21 |
|  |  |  | Z1 | 15 |
|  |  |  | Z2 | 15 |
|  |  | L5 | V1 | 12 |
|  |  |  | Z1 | – |
|  |  |  | Z2 | – |
| N022 | >45 years | L3 | V1 | 14 |
|  |  |  | Z1 | 10 |
|  |  |  | Z2 | 10 |
|  |  | L4 | V1 | 13 |
|  |  |  | Z1 | 12 |
|  |  |  | Z2 | 12 |
|  |  | L5 | V1 | 11 |
|  |  |  | Z1 | 11 |
|  |  |  | Z2 | 11 |
| N033 | 25–45 years | L3 | V1 | 10 |
|  |  |  | Z1 | 10 |
|  |  |  | Z2 | 10 |
|  |  | L4 | V1 | 10 |
|  |  |  | Z1 | – |
|  |  |  | Z2 | – |
|  |  | L5 | V1 | 10 |
|  |  |  | Z1 | – |
|  |  |  | Z2 | – |
| N035 | >45 years | L3 | V1 | 15 |
|  |  |  | Z1 | – |
|  |  |  | Z2 | 15 |
|  |  | L4 | V1 | 15 |
|  |  |  | Z1 | 15 |
|  |  |  | Z2 | 15 |
|  |  | L5 | V1 | 15 |
|  |  |  | Z1 | 11 |
|  |  |  | Z2 | – |
| N039 | 25–45 years | L3 | V1 | 15 |
|  |  |  | Z1 | 15 |
|  |  |  | Z2 | 15 |
|  |  | L4 | V1 | 15 |
|  |  |  | Z1 | 15 |
|  |  |  | Z2 | 15 |
|  |  | L5 | V1 | 15 |
|  |  |  | Z1 | – |
|  |  |  | Z2 | – |
| M059 | 25–45 years | L3 | V1 | 12 |
|  |  |  | Z1 | 12 |
|  |  |  | Z2 | 12 |
|  |  | L4 | V1 | 12 |
|  |  |  | Z1 | 13 |
|  |  |  | Z2 | 12 |
|  |  | L5 | V1 | 14 |
|  |  |  | Z1 | 15 |
|  |  |  | Z2 | 15 |
| M068 | 18–25 years | L3 | V1 | 12 |
|  |  |  | Z1 | 12 |
|  |  |  | Z2 | 13 |
|  |  | L4 | V1 | 12 |
|  |  |  | Z1 | 12 |
|  |  |  | Z2 | 12 |
|  |  | L5 | V1 | 12 |
|  |  |  | Z1 | 12 |
|  |  |  | Z2 | 12 |
| M148 | 18–25 years | L3 | V1 | 10 |
|  |  |  | Z1 | 10 |
|  |  |  | Z2 | 11 |
|  |  | L4 | V1 | 13 |
|  |  |  | Z1 | 12 |
|  |  |  | Z2 | 10 |
|  |  | L5 | V1 | 11 |
|  |  |  | Z1 | 12 |
|  |  |  | Z2 | 12 |
| M160 | 18–25 years | L3 | V1 | 21 |
|  |  |  | Z1 | 13 |
|  |  |  | Z2 | 11 |
|  |  | L4 | V1 | 12 |
|  |  |  | Z1 | 13 |
|  |  |  | Z2 | 12 |
|  |  | L5 | V1 | 13 |
|  |  |  | Z1 | 16 |
|  |  |  | Z2 | 12 |
